# The inflammatory microenvironment repurposes BMP-2 signaling to drive pathological osteophyte formation in osteoarthritis

**DOI:** 10.3389/fimmu.2026.1776833

**Published:** 2026-03-25

**Authors:** Chao Yu, Jun Qin, Xi Zhao, Ruifu Li, Zili Zeng, Hao Zeng, Yongyong Li, Liang Guo

**Affiliations:** 1 Department of Orthopaedic Surgery, The University-Town Hospital of Chongqing Medical University, Chongqing, China; 2 Medical Sciences Research Center, University-Town Hospital of Chongqing Medical University, Chongqing, China; 3 Molecular Oncology Laboratory, Department of Orthopedic Surgery and Rehabilitation Medicine, The University of Chicago Medical Center, Chicago, IL, United States; 4 Department of Gerontology, The Second Affiliated Hospital of Chongqing Medical University, Chongqing, China; 5 Department of Bone and Soft Tissue Tumor, Chongqing University Cancer Hospital, Chongqing, China

**Keywords:** BMP-2, chondrocyte hypertrophy, inflammation, interleukin-1 beta, microenvironment, osteoarthritis, osteophyte formation

## Abstract

**Background:**

Osteoarthritis (OA) is increasingly recognized as a low-grade inflammatory disease, yet how the inflammatory milieu interacts with regenerative signals remains poorly understood. Bone morphogenetic protein 2 (BMP-2) typically functions as an anabolic factor for cartilage; however, its levels are paradoxically elevated in degenerating joints. This study investigates whether the inflammatory microenvironment acts as a molecular switch that alters the functional outcome of BMP-2 signaling from repair to pathology.

**Methods:**

BMP-2 levels were quantified in human OA cartilage and synovial fluid via immunofluorescence and ELISA, and correlated with radiographic severity (Kellgren-Lawrence grade). To model the inflammatory niche, ATDC5 chondrocytes and bone marrow–derived mesenchymal stem cells (BMSCs) were co-stimulated with BMP-2 and Interleukin-1 beta (IL-1β). *In vivo*, a murine OA model was induced by destabilization of the medial meniscus (DMM), followed by intra-articular delivery of adenoviral vectors encoding BMP-2 and/or IL-1β to assess joint pathology and osteophyte formation.

**Results:**

Clinical analysis revealed that BMP-2 expression is significantly upregulated in OA tissues and correlates positively with disease severity. Mechanistically, IL-1β stimulation induced endogenous BMP-2 expression in chondrocytes but blocked its chondrogenic effects, instead promoting hypertrophy. Crucially, the combination of BMP-2 and IL-1β synergistically amplified osteogenic differentiation in BMSCs, as evidenced by robust upregulation of *Runx2*, *OPN*, and *OCN*. *In vivo*, while BMP-2 alone showed mild protective effects, its presence in an IL-1β-rich environment severely exacerbated joint destruction and triggered massive osteophyte formation.

**Conclusion:**

Inflammation fundamentally repurposes BMP-2 signaling in the OA joint. Instead of promoting homeostasis, the synergy between BMP-2 and inflammatory cytokines drives mesenchymal progenitors toward pathological ossification. These findings highlight the critical role of the inflammatory microenvironment in specific OA phenotypes in dictating tissue fate and suggest that targeting this inflammatory switch is essential for effective OA therapy.

## Introduction

Osteoarthritis (OA) represents a failure of joint homeostasis, manifesting not only as the progressive erosion of articular cartilage but also as the aberrant formation of osteophytes—bony outgrowths at the joint margins (1, 2). While cartilage loss has traditionally been the primary focus of OA research, osteophytes are a hallmark of structural progression and a major source of pain and functional limitation (3). The development of osteophytes involves the reactivation of endochondral ossification, a process normally restricted to skeletal development and fracture repair. However, the specific molecular signals that trigger this pathological “repair” response within the chronic inflammatory microenvironment of the OA joint remain paradoxically defined.

Bone morphogenetic protein 2 (BMP-2), a potent member of the transforming growth factor-beta (TGF-β) superfamily, sits at the center of this paradox (4). Under physiological conditions, BMP-2 is an essential anabolic factor, critical for mesenchymal condensation and chondrogenic differentiation (5). Based on these properties, exogenous BMP-2 has been widely explored as a therapeutic candidate for cartilage regeneration. However, clinical and experimental observations paint a conflicting picture: BMP-2 expression is frequently upregulated in OA cartilage and synovial fluid, correlating with disease severity rather than repair (6, 7). Furthermore, while mechanical stress and pro-inflammatory cytokines (e.g., IL-1β, TNF-α) can induce BMP-2 expression in chondrocytes (8, 9), this endogenous upregulation fails to halt degeneration and may instead fuel chondrocyte hypertrophy and ectopic ossification (10).

This duality suggests that BMP-2 does not act in isolation; rather, its functional outcome is strictly dictated by the local microenvironment (11, 12). In the OA joint, cartilage injury recruits mesenchymal progenitor cells from the synovium and marrow space to participate in tissue repair (13, 14). We postulate that the concurrent presence of chronic inflammation—a phenotype increasingly recognized in OA subsets—fundamentally alters the response of these progenitors to BMP-2. Instead of promoting hyaline cartilage regeneration, we hypothesize that the inflammatory milieu “repurposes” BMP-2 signaling, diverting these cells toward a pathological osteogenic fate (15, 16). This maladaptive synergy could explain why attempting to boost regenerative signaling in an inflamed joint often accelerates osteophyte formation rather than restoring cartilage integrity.

To dissect this context-dependent mechanism, this study integrates clinical data with mechanistic *in vitro* and *in vivo* models. We first quantified BMP-2 levels in human OA specimens to establish clinical relevance. We then utilized chondrocytes and mesenchymal stem cells to delineate the synergistic interplay between BMP-2 and IL-1β. Finally, employing a murine model of instability-induced OA, we demonstrate that the addition of an inflammatory stimulus transforms BMP-2 from a benign or potentially reparative factor into a potent driver of joint destruction and osteophyte progression. This study adheres to the ARRIVE guidelines for the reporting of animal experiments (17).

## Materials and methods

### Human sample collection

Synovial fluid and cartilage tissues were obtained from 12 patients (range from 47 to 65 years old, 7 females and 5 males) diagnosed with knee OA (Kellgren-Lawrence [K-L] grades 3–4) undergoing total knee arthroplasty at The University-town Hospital of Chongqing Medical University. 12 control samples were collected from individuals with no history of OA undergoing arthroscopic surgery for acute ligamentous injuries. Informed consent was obtained from all participants. The study protocol was approved by the Institutional Ethics Committee (No. 2021.5.28/LL-202133) and conducted in accordance with the Declaration of Helsinki.

### Immunohistochemistry and immunofluorescence

Human cartilage specimens were fixed in 4% paraformaldehyde, decalcified, embedded in paraffin, and sectioned at 5 μm. Sections underwent antigen retrieval and were incubated with primary antibodies against BMP-2 (1:200; Abcam, Cambridge, UK) and Collagen Type X (Col-X; 1:200; Cell Signaling Technology, Danvers, MA, USA). For immunohistochemistry (IHC), detection was performed using a DAB substrate kit. For immunofluorescence (IF), sections were incubated with Alexa Fluor 488- or 594-conjugated secondary antibodies (1:500; Invitrogen), and nuclei were counterstained with DAPI. Images were captured using a fluorescence microscope (Olympus, Tokyo, Japan).

### Enzyme-linked immunosorbent assay

BMP-2 concentrations in synovial fluid were quantified using a human BMP-2 ELISA kit (Shanghai Xinyu Biotechnology Co., Ltd., China) following the manufacturer’s protocol. Synovial fluid samples were centrifuged at 3,000 rpm for 10 minutes at 4 °C to remove cellular debris prior to analysis. Absorbance was measured at 450 nm using a microplate reader (BioTek, Winooski, VT, USA).

### Cell culture and treatment

The murine chondrogenic cell line ATDC5 and primary bone marrow–derived mesenchymal stem cells (BMSCs) were utilized. Cells were cultured in DMEM/F12 (Gibco) supplemented with 10% fetal bovine serum (FBS) and 1% penicillin/streptomycin. To simulate an inflammatory environment, ATDC5 cells were treated with recombinant mouse IL-1β (10 ng/mL; PeproTech) and/or BMP-2 (100 ng/mL; R&D Systems) for 72 hours. BMSCs were transduced with adenoviral vectors as described below. Gene expression was analyzed via RT-qPCR.

### Real-time quantitative PCR

Total RNA was extracted using TRIzol reagent (Invitrogen). cDNA was synthesized using a PrimeScript RT Master Mix (Takara, Japan). qPCR was performed using SYBR Green Master Mix (Roche) on a LightCycler 480 system. Relative gene expression was calculated using the 2^-ΔΔCt^ method, normalized to *Gapdh*. Primers targeted *Bmp2*, *Col10a1*, *Alp*, *Runx2*, and *Mmp13*. The specific primer sequences used in this study are listed in **Table 1**.

**Table 1:** Primer Sequences Used for qRT-PCR.

Gene	Forward Primer (5’–3’)	Reverse Primer (5’–3’)
BMP-2	AGCTTCCACCATGATGAATCTACA	TTCAGGTTGAAGAGGAACCGC
COL2A1	GGCAATAGCAGGTTCACGTACA	CGATAACAGTCTTGCCCCACTT
COL10A1	CCATGTGTTTGCACTGCCA	GGGATGTCTTGGATAACCTCTTG
Aggrecan	TGTGGTTCTGGGAGTTGGAAGT	AGGTCTGAAGCACGTTGTCATT
MMP13	CCTGGAATTGGCAACAAAGTGA	TCGGAGACTGGTAATGGCAGT
RANKL	TATAGAATCCCATCCCAGCCTTT	AGGTTAGTGAGGTCCAGTTAGC
OCN	CACTCCTCGCCCTATTGGC	CCCTCCTGCTTGGACACAAA
OPN	AGACACATATGATGGCCGAGGT	TGGCTGTCCACCTTGTAGACTC
ALP	TGCTGGACCTCGTTGACAC	ACTTCCCCAGGAGAGTCGTT
RUNX2	CCGCCTCAGTGATTTAGGGC	GGGTCTGTAATCTGACTCTGTCC
GAPDH	AGGTCGGTGTGAACGGATTTG	TGTAGACCATGTAGTTGAGGTCA

### Alkaline phosphatase and Alizarin Red S staining

Osteogenic differentiation of BMSCs was assessed after 7 and 14 days of induction. ALP activity was visualized using an ALP staining kit (Beyotime, Shanghai, China). Mineralized matrix deposition was evaluated using Alizarin Red S staining (Solarbio, Beijing, China). Quantification was performed by measuring absorbance after solubilization.

### Adenoviral vector construction

Recombinant adenoviruses encoding mouse BMP-2 (Ad-BMP-2, GFP-tagged) and IL-1β (Ad-IL-1β, RFP-tagged) were constructed by the Molecular Oncology Laboratory at the University of Chicago. An empty vector expressing GFP (Ad-GFP) served as the control.

### *In vivo* ectopic bone formation assay

To assess osteogenic potential *in vivo*, BMSCs transduced with Ad-BMP-2, Ad-IL-1β, or their combination were resuspended in PBS and injected subcutaneously into the flanks of 6-week-old BALB/c nude mice (n=5 per group). Prior to cell implantation, mice were anesthetized via inhalation of 2% isoflurane in oxygen. Implants were harvested at 8 weeks for histological analysis (H&E and Masson’s trichrome staining). For sample collection, mice were euthanized by CO_2_ inhalation (flow rate of 30–70% of the chamber volume per minute) followed by cervical dislocation to ensure death.

### Mouse osteoarthritis model

All animal procedures were approved by the Animal Care and Use Committee of Chongqing Medical University. Eight-week-old male C57BL/6 mice were subjected to surgical destabilization of the medial meniscus (DMM) to induce OA (18). Mice were anesthetized with an intraperitoneal injection of sodium pentobarbital (50 mg/kg) during the surgery and intra-articular injections. Mice were randomly assigned to four groups (n=5 per group): (1) Control (DMM + Ad-GFP), (2) Ad-BMP-2, (3) Ad-IL-1β, and (4) Ad-BMP-2 + Ad-IL-1β. A single intra-articular injection of the respective adenovirus (1×10^8^ PFU in 10 μL) was administered one week post-surgery. Mice were sacrificed at 8 weeks post-injection. Euthanasia was performed by intraperitoneal injection of an overdose of sodium pentobarbital (150 mg/kg), followed by cervical dislocation to confirm death.

### Micro-CT and histological analysis

Knee joints were fixed in 4% paraformaldehyde and scanned using a micro-CT system (Scanco Medical, Switzerland) at 70 kVp, 114 μA, with an isotropic voxel size of 10 μm to evaluate osteophyte volume. Images were reconstructed using the Scanco Evaluation Program (v6.6) with a Gaussian filter (sigma = 0.8, support = 1) and a threshold of 220–1000 Scanco units (~350 mg HA/cm³). Osteophytes were identified as ectopic bone formations at the joint margins, segmented semi-automatically, and their volumes were quantified using voxel-based morphometry. Three-dimensional reconstructions were generated using Amira software (v2020.2) for visualization.

OA severity was graded using the OARSI scoring system (19). Osteophyte formation was evaluated on Safranin O/Fast Green stained sections using a standardized scoring system. Osteophyte size was scored as: 0 = none, 1 = small (≤ cartilage thickness), 2 = medium (1-3× cartilage thickness), 3 = large (>3× cartilage thickness). Osteophyte maturity was scored as: 0 = none, 1 = cartilaginous (Col-X+, proteoglycan-rich), 2 = mixed (partial endochondral ossification), 3 = bony (mature bone with marrow). A composite score (size + maturity, range 0-6) was calculated. Scoring was performed by two blinded observers on 5–6 sections per joint at 60-90 μm intervals. Immunohistochemistry for Col-X was performed to evaluate chondrocyte hypertrophy.

### Statistical analysis

Data are presented as mean ± standard deviation (SD). Individual data points are overlaid on bar graphs to show data distribution. Sample sizes (n) are indicated in each figure legend and represent biological replicates unless otherwise specified. Statistical differences between multiple groups were analyzed using one-way or two-way analysis of variance (ANOVA) followed by Tukey’s *post hoc* test. Correlation analysis was performed using Pearson’s correlation coefficient. A *p*-value < 0.05 was considered statistically significant. Analysis was conducted using GraphPad Prism 9.0 software.

## Results

### Elevated BMP-2 expression correlates with disease severity in human OA

**Figure 1:**
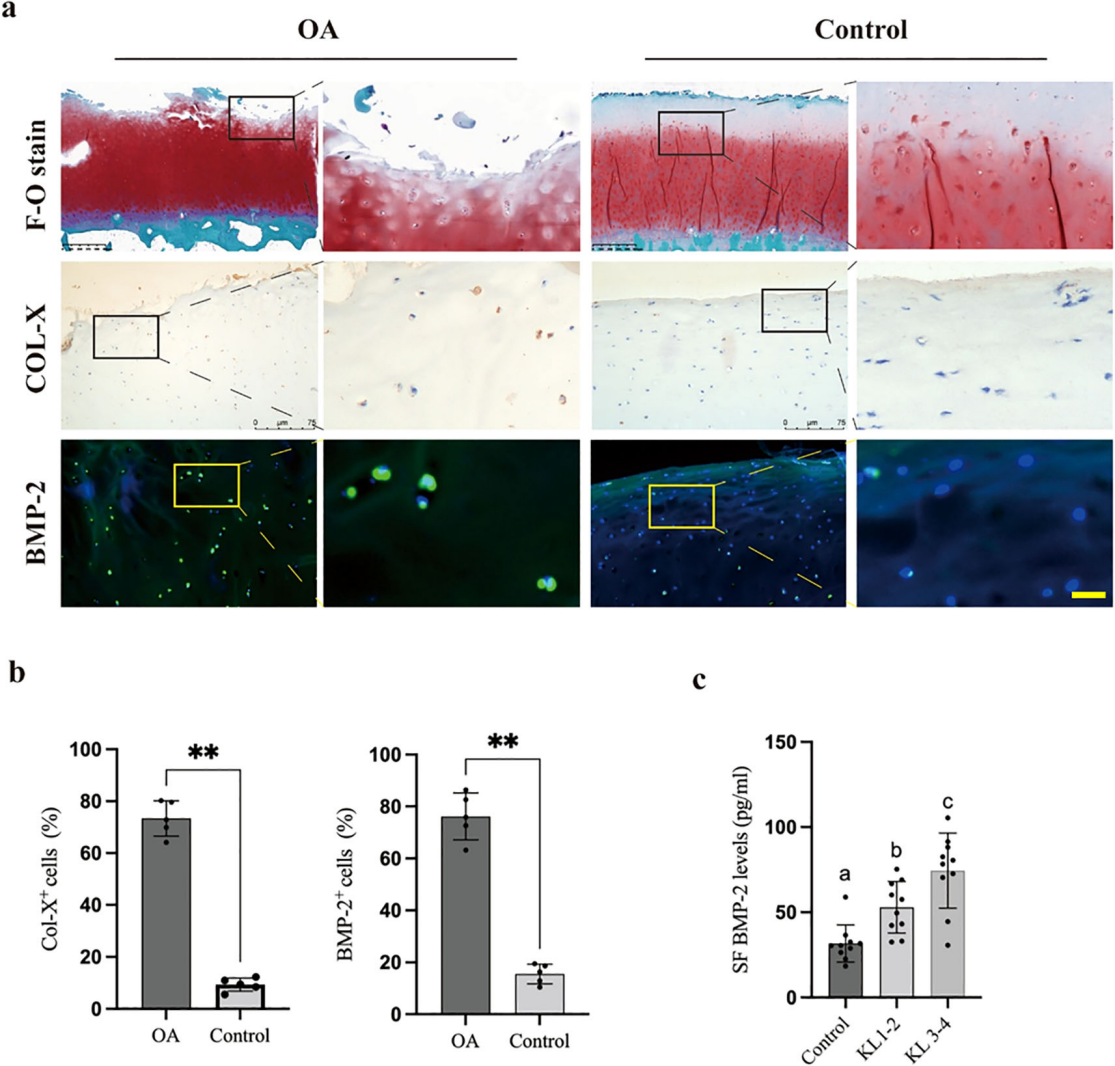
BMP-2 expression is upregulated in human OA cartilage and synovial fluid. **(a)** Safranin O/Fast Green staining (top), Immunohistochemistry for Col-X (middle), and Immunofluorescence for BMP-2 (bottom) in human normal and OA cartilage sections. Scale bar = 100 μm. **(b)** Quantification of the percentage of Col-X and BMP-2 positive cells. **(c)** ELISA quantification of BMP-2 levels in synovial fluid from healthy controls and OA patients with varying K-L grades. Data are mean ± SD. **p < 0.01 vs. Control/Healthy; ##p < 0.01 vs. K-L 1-2.

To establish the clinical relevance of BMP-2 in OA, we analyzed human cartilage and synovial fluid samples. Safranin O/Fast Green staining confirmed severe cartilage matrix loss in OA samples compared to healthy controls (**Figure 1a**). Immunohistochemical analysis revealed a marked accumulation of Col-X positive hypertrophic chondrocytes in OA cartilage. Notably, immunofluorescence staining demonstrated significantly elevated BMP-2 protein levels in the OA cartilage matrix compared to controls (**Figure 1a**). Quantitative analysis confirmed a significant increase in the percentage of both Col-X and BMP-2 positive cells in OA tissues (p < 0.01; **Figure 1b**).

Furthermore, ELISA analysis of synovial fluid (SF) revealed that BMP-2 concentrations were significantly higher in OA patients compared to healthy controls. Importantly, SF BMP-2 levels exhibited a positive correlation with radiographic disease severity, with K-L grade 3–4 patients showing significantly higher levels than those with K-L grade 1-2 (**Figure 1c**). These data suggest that BMP-2 upregulation is a pathological feature closely associated with the progression of human OA.

### Inflammatory cytokines induce BMP-2 and impair its chondrogenic function

**Figure 2:**
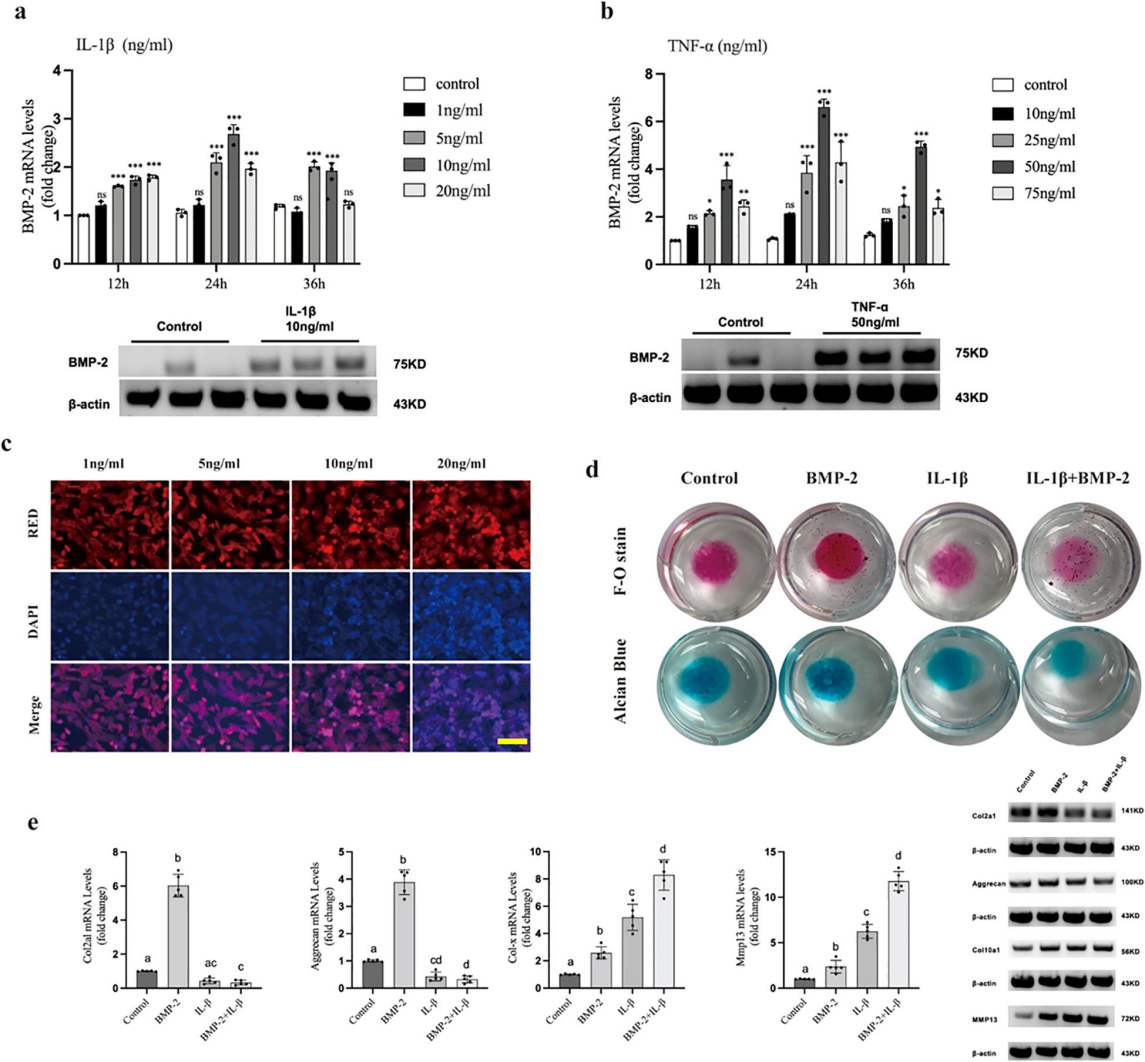
Inflammation induces BMP-2 expression but impairs its anabolic effects in chondrocytes. **(a, b)** RT-qPCR analysis of BMP-2 mRNA levels and Western Blot analysis of BMP-2 protein levels in ATDC5 cells stimulated with IL-1β or TNF-α at indicated doses and times. **(c)** DHE staining showing ROS production induced by IL-1β. Scale bar = 50 μm. **(d)** Safranin O and Alcian Blue staining of ATDC5 micromasses treated with BMP-2, IL-1β, or both. **(e)** RT-qPCR analysis of chondrogenic (Col2a1, Aggrecan), hypertrophic (Col10a1), and catabolic (Mmp13) markers. Data are mean ± SD. *p < 0.05, **p < 0.01, ***p < 0.001. Experiments was repeated three times.

We next investigated the regulation of BMP-2 by inflammatory stimuli. Stimulation of ATDC5 chondrocytes with IL-1β or TNF-α resulted in a dose- and time-dependent upregulation of BMP-2 mRNA expression (**Figures 2a, b**). Under the optimal stimulation conditions (10 ng/ml IL-1β and 50 ng/ml TNF-α), the expression level of BMP-2 protein in the ATDC5 chondrocytes significantly increased after 24 hours of treatment (**Figures 2a, b**). IL-1β (10 ng/mL), which also induced significant oxidative stress (**Figure 2c**), was selected for subsequent experiments to mimic the inflamed joint environment.

While treatment with BMP-2 alone promoted proteoglycan synthesis (indicated by intense Safranin O and Alcian blue staining), this anabolic effect was significantly blunted in the presence of IL-1β (**Figure 2d**). Gene expression analysis revealed that IL-1β not only suppressed BMP-2-induced expression of chondrogenic markers (*Col2a1*, *Aggrecan*) but also synergistically upregulated hypertrophic (*Col10a1*) and catabolic (*Mmp13*) markers (**Figure 2e**). Under the stimulation of IL-1β and BMP-2, the protein expression levels of *Col2a1, Aggrecan, Col10a1* and *Mmp13* in ATDC5 chondrocytes were consistent with those of their corresponding mRNAs (**Figure 2e**). These findings indicate that the reparative capacity of BMP-2 is compromised under inflammatory conditions, shifting chondrocytes toward a hypertrophic and catabolic phenotype.

### Synergistic promotion of osteogenesis by BMP-2 and IL-1β in BMSCs

**Figure 3:**
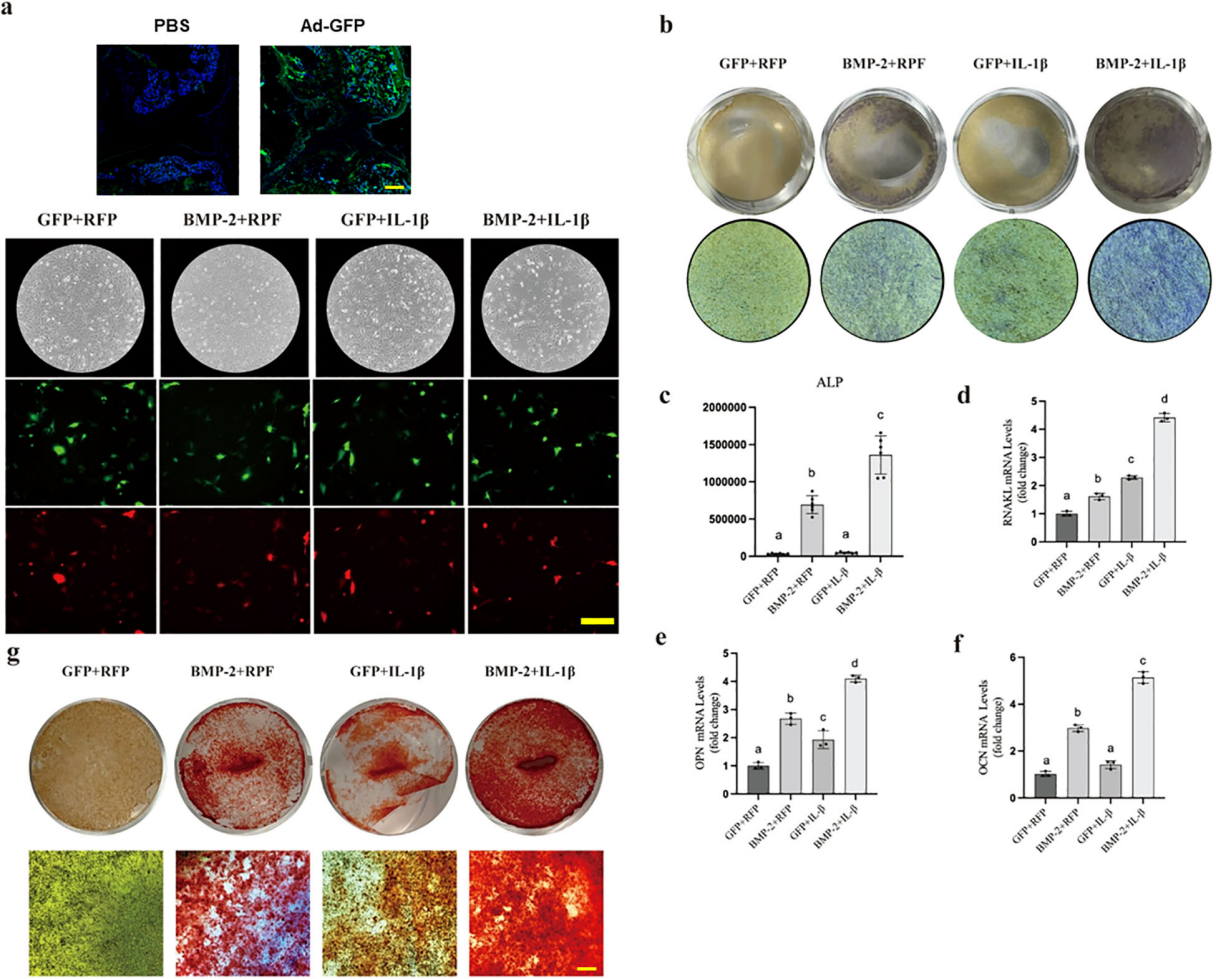
BMP-2 and IL-1β synergistically promote osteogenic differentiation of BMSCs. **(a)** Fluorescence microscopy of BMSCs transduced with Ad-BMP-2 (GFP) and Ad-IL-1β (RFP). **(b)** ALP staining at Day 7. **(c)** Quantification of ALP activity. **(d-f)** RT-qPCR analysis of osteogenic markers RANKL, OPN, and OCN. **(g)** Alizarin Red S staining for mineralization at Day 14. Data are mean ± SD. Different letters (a, b, c, d, e) indicate statistically significant differences between groups (p < 0.01). Experiments were repeated three times. Scale bar = 50 µm.

Given that mesenchymal progenitors in the joint contribute to osteophyte formation, we examined the effect of BMP-2 and IL-1β on BMSCs. Adenovirus transfection and co-transduction efficiency were confirmed via fluorescence microscopy (**Figure 3a**). While BMP-2 overexpression alone enhanced ALP activity, the addition of IL-1β significantly amplified this osteogenic response (**Figures 3b, c**). Consistently, the combination of BMP-2 and IL-1β led to a robust upregulation of osteoclast differentiation regulator (*RANKL*) and osteogenic transcription factors(*Opn*, and *Ocn*), compared to BMP-2 alone (**Figures 3c–e**). Alizarin Red S staining further confirmed that mineralization was maximally enhanced in the combined treatment group (**Figure 3g**). These *in vitro* data suggest a synergistic interaction between osteogenic (BMP-2) and inflammatory (IL-1β) signals in driving mesenchymal differentiation toward bone.

### BMP-2 and IL-1β accelerate ectopic bone formation *in vivo*

**Figure 4:**
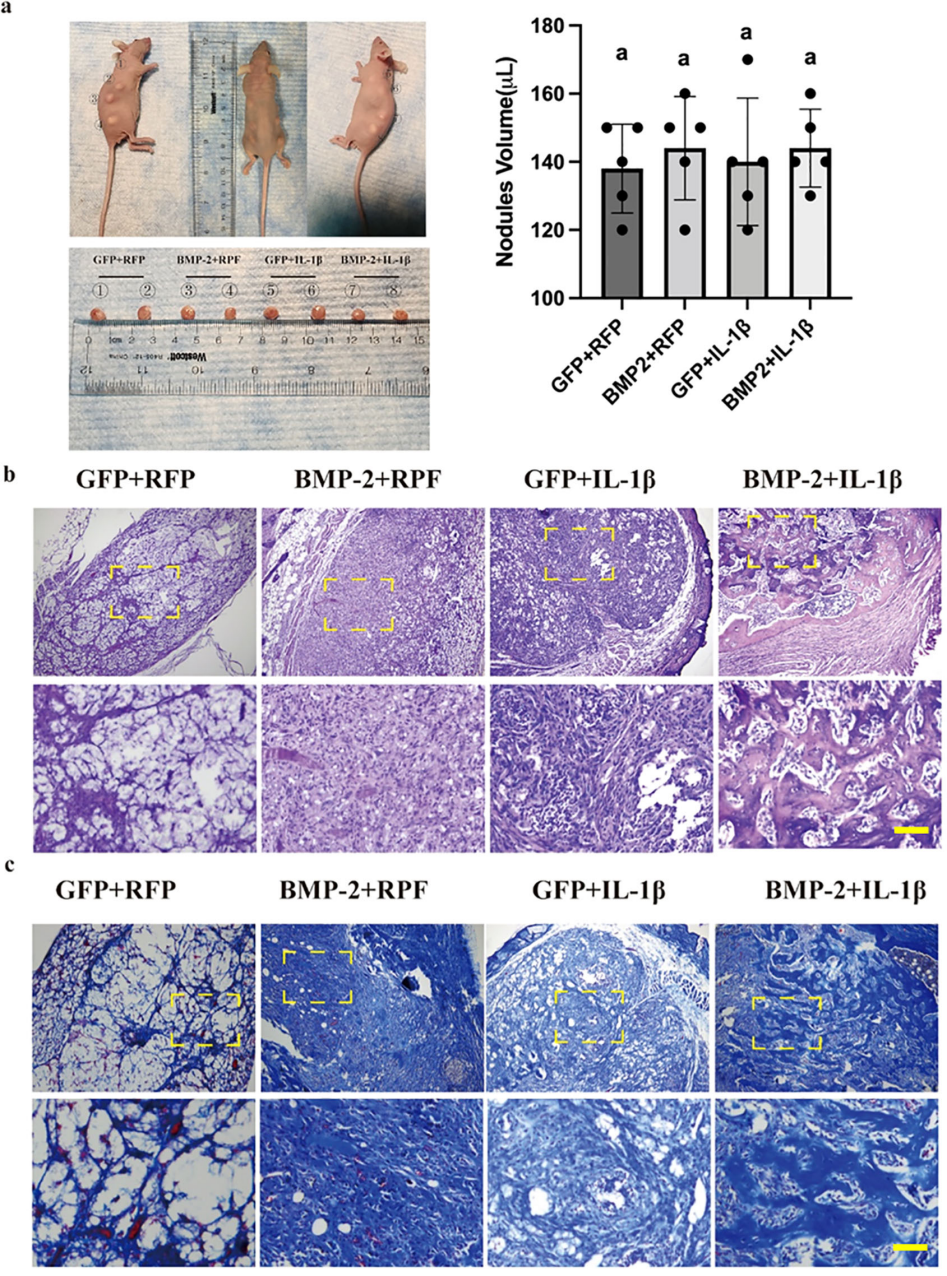
Synergistic induction of ectopic bone formation *in vivo*. **(a)** Gross appearance of subcutaneous nodules harvested from nude mice at 8 weeks. **(b)** H&E staining showing osteoid formation (arrows). **(c)** Masson’s trichrome staining showing collagen deposition (blue). Note the mature bone formation in the BMP-2 + IL-1β group. n=5. Scale bar = 50 μm.

To validate the osteogenic synergy *in vivo*, transduced BMSCs were implanted subcutaneously into nude mice. While gross nodule size was comparable across groups (**Figure 4a**), histological evaluation revealed distinct differences in tissue composition. The Control (GFP+RFP) and IL-1β-only groups showed minimal osteoid formation. The BMP-2 group exhibited moderate bone formation. However, the BMP-2 + IL-1β group displayed extensive, mature trabecular bone-like structures with dense collagen deposition, as evidenced by H&E and Masson’s trichrome staining (**Figures 4b, c**). This confirms that the presence of an inflammatory stimulus significantly potentiates BMP-2-mediated osteogenesis *in vivo*.

### Inflammation transforms BMP-2 into a driver of osteophyte formation and OA progression

**Figure 5:**
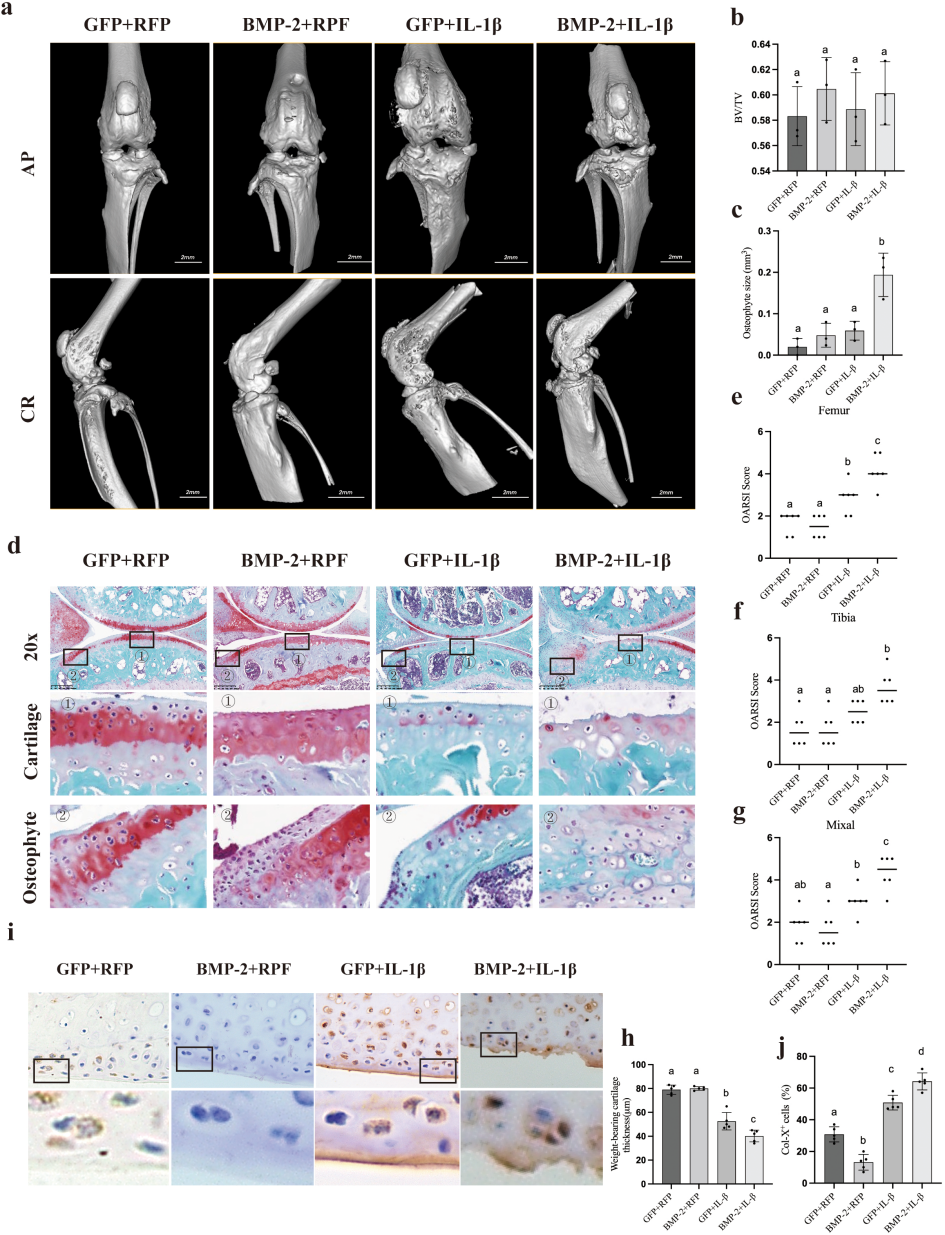
BMP-2 exacerbates osteophyte formation and OA progression under inflammatory conditions in the DMM model. **(a)** Representative micro-CT 3D reconstructions of knee joints. Arrows indicate osteophytes. **(b, c)** Quantification of osteophyte volume. **(d)** Safranin O/Fast Green staining of articular cartilage. **(e)** OARSI scoring of cartilage degeneration. **(f-h)** Detailed histological scoring and cartilage thickness. **(i)** Immunohistochemistry for Col-X in cartilage sections. **(j)** Quantification of Col-X positive area. Comparison groups include Control (DMM+Ad-GFP), Ad-BMP-2, Ad-IL-1β, and Combined. Data are mean ± SD, n = 5. Different letters (a, ab, b, c, d) and symbols (#) indicate statistically significant differences between groups (p < 0.01). Scale bar = 100 µm.

Finally, we evaluated the impact of BMP-2 in the DMM-induced mouse OA model. Micro-CT reconstruction at 8 weeks post-surgery revealed that mice co-injected with Ad-BMP-2 and Ad-IL-1β developed significantly larger osteophytes at the medial joint margins compared to the Control (DMM + Ad-GFP) and Ad-BMP-2 only groups (**Figures 5a–c**). Histological analysis showed that while Ad-BMP-2 alone conferred a mild protective effect on cartilage integrity, the combination with Ad-IL-1β resulted in severe cartilage erosion and proteoglycan loss (**Figure 5d**). OARSI scores were highest in the combined treatment group, indicating exacerbated disease progression (**Figures 5e–g**).

Immunohistochemistry for Col-X confirmed that the synergistic treatment markedly increased chondrocyte hypertrophy in the deep and calcified cartilage zones (**Figures 5i, j**). Collectively, these results demonstrate that in an inflamed joint, BMP-2 exacerbates osteophyte formation and cartilage degeneration.

## Discussion

This study provides a novel and nuanced perspective on the role of BMP-2 in osteoarthritis, reconciling its paradoxical functions as both a reparative agent and a pathogenic driver. We demonstrate that while BMP-2 expression is consistently elevated in OA tissues and correlates with disease severity, its biological outcome is critically dictated by the inflammatory microenvironment. Our central finding is that inflammation serves as a molecular switch: in its absence, BMP-2 functions anabolically; in its presence (specifically high IL-1β), BMP-2 is repurposed to fuel pathological osteophyte formation and accelerate cartilage hypertrophy.

The elevated levels of BMP-2 in OA synovial fluid, which we found to correlate strongly with radiographic severity, likely reflect a compensatory but maladaptive response to tissue injury. This aligns with the “wound healing” theory of OA, where the joint attempts to repair cartilage defects but fails due to the hostile inflammatory milieu. Our *in vitro* data revealed a feed-forward loop wherein IL-1β stimulates chondrocytes to produce more BMP-2. However, rather than repairing the matrix, this excess BMP-2—in the context of sustained inflammation—fails to upregulate hyaline cartilage markers (*Col2a1, Aggrecan*) and instead promotes hypertrophic differentiation (*Col10a1, Mmp13*). This suggests that inflammation uncouples the chondrogenic activity of BMP-2 from its osteogenic potential, biasing the cell fate toward terminal differentiation and calcification.

A key mechanistic insight from our study is the profound synergy between BMP-2 and IL-1β in driving osteogenesis in mesenchymal progenitors. This challenges the traditional view that inflammation is purely catabolic. We hypothesize that this synergy stems from crosstalk between the NF-κB and Smad signaling pathways. While BMP-2 canonically activates Smad1/5/8 to induce osteoblastogenesis (4, 20, 21), pro-inflammatory cytokines activate NF-κB and MAPK pathways (22, 23). Recent evidence suggests that NF-κB activation can enhance the stability or transcriptional activity of Runx2, the master osteogenic regulator, thereby sensitizing cells to BMP signaling (23, 24). This molecular convergence explains why osteophytes grow rapidly in inflammatory OA phenotypes despite the catabolic environment: the inflammatory signals prime the progenitors to overreact to the available BMP-2. It is important to note that while RANKL is classically recognized as the master regulator of osteoclast differentiation (25), its upregulation in our BMSC differentiation model likely reflects the coupling mechanism between bone formation and resorption. In physiological bone remodeling, osteoblast-derived RANKL promotes osteoclastogenesis to facilitate the resorption phase, which is subsequently followed by new bone formation (26). In the context of pathological osteophyte formation, the aberrant upregulation of both osteogenic markers (Runx2, OPN, OCN) and RANKL suggests an imbalance in the coupling process, potentially leading to the disorganized bone architecture characteristic of osteophytes.

Our findings have significant clinical implications, particularly for “regenerative medicine” approaches in OA. The application of BMP-2 or other growth factors has been proposed for cartilage repair (11, 27). However, our DMM model data serves as a cautionary tale: intra-articular BMP-2 administration in the presence of IL-1β (mimicking active synovitis) led to disastrous joint destruction and massive osteophytosis, consistent with evidence that BMP-2 can induce significant osteophyte formation (6, 28). This helps explain the inconsistency observed in clinical trials and suggests that a “one-size-fits-all” approach is flawed. We propose that OA patients must be stratified based on their synovial inflammatory status. For patients with “inflammatory OA” (high IL-1β/TNF-α), BMP-2 therapy would be contraindicated unless preceded by robust anti-inflammatory conditioning. Combinatorial therapies targeting both the inflammatory axis (e.g., IL-1Ra) and the regenerative axis (e.g., BMP-2) may represent a more viable strategy to unlock the reparative potential of BMPs without triggering ectopic bone formation, as suggested by studies on combined gene therapy approaches (29).

We must acknowledge several limitations to facilitate a balanced interpretation of these data. First, while we posit that the synovial BMP-2 is “chondrocyte-derived” based on the strong correlation with Col-X+ chondrocytes, we cannot definitively exclude contributions from synoviocytes or macrophages without lineage-tracing models. Nevertheless, the clinical correlation remains valid regardless of the cellular source. Second, we utilized BMSCs as a proxy for the intra-articular mesenchymal progenitor pool. While BMSCs and synovial MSCs share osteogenic plasticity (13, 30), synovial MSCs may have distinct migratory and responsiveness profiles relevant to osteophyte formation. Third, the use of adenoviral vectors induces supraphysiological cytokine levels that are sustained, whereas human OA involves chronic, fluctuating low-grade inflammation (31, 32). Despite these limitations, the robust phenotype observed across both *in vitro* and *in vivo* models provides compelling evidence for the inflammation-dependent duality of BMP-2.

This study has the following limitations: the adenovirus vector-induced IL-1β level is supraphysiological, creating an acute and high-intensity inflammatory microenvironment, which is different from the low-grade and fluctuating inflammatory characteristics of clinical OA; the DMM model is post-traumatic OA, which progresses rapidly and differs from the slow course of human primary OA. Therefore, this conclusion is most applicable to inflammatory OA and the acute phase of post-traumatic OA, and may not be applicable to non-inflammatory OA or patients in the chronic stable phase. Future studies need to use chronic low-grade inflammation models (such as transgenic mice) and humanized models for further verification.

**Figure 6:**
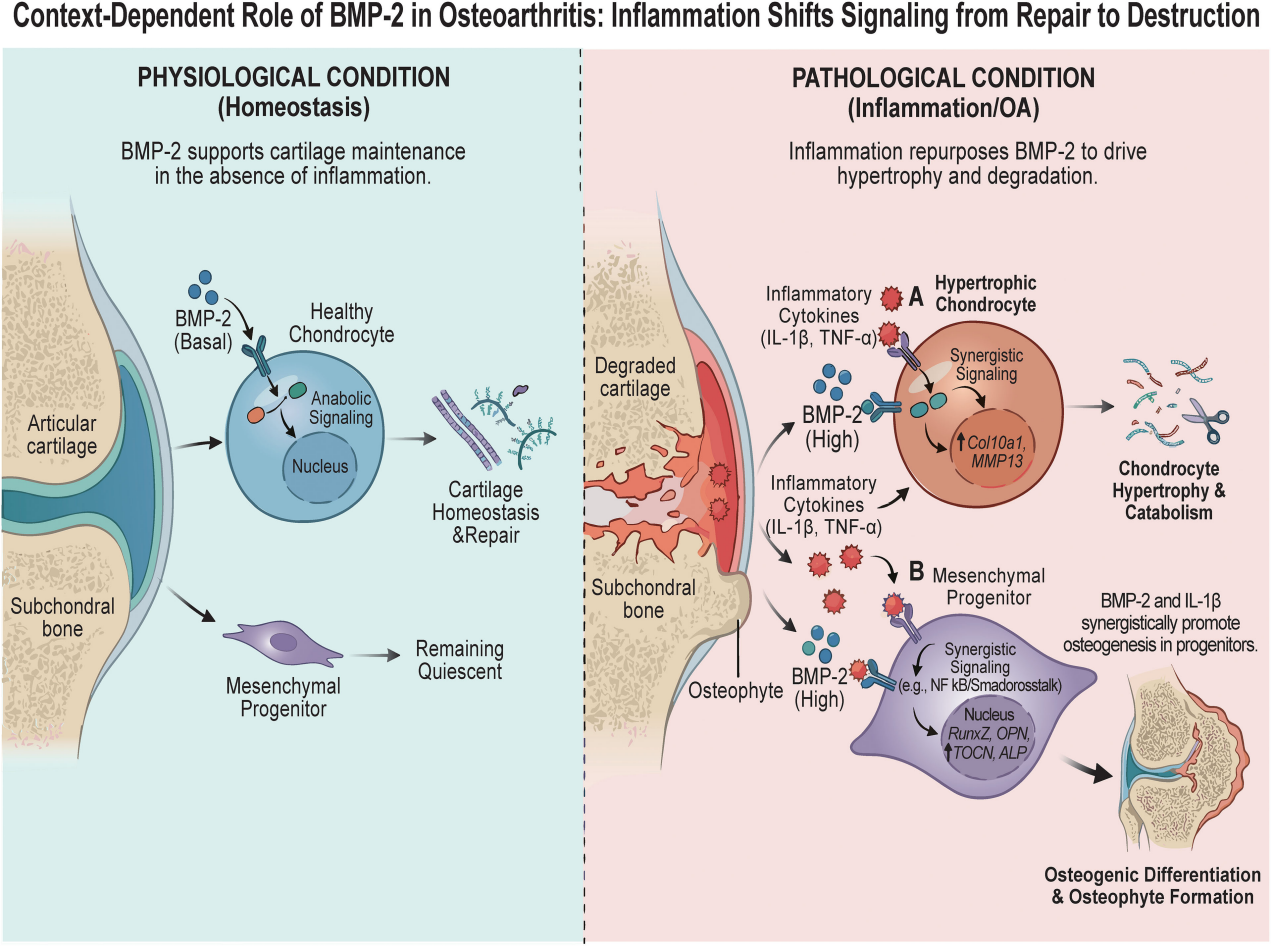
Schematic diagram illustrating the context-dependent role of BMP-2 in osteoarthritis: inflammation shifts signaling from repair to destruction. Under physiological conditions (left), basal BMP-2 signaling functions as an anabolic factor to support articular cartilage maintenance and repair while mesenchymal progenitors remain quiescent. In contrast, within the pathological inflammatory microenvironment of OA (right), BMP-2 signaling is repurposed to drive joint degeneration. Specifically, inflammatory cytokines synergize with high levels of BMP-2 to promote chondrocyte hypertrophy and catabolism **(A)**, characterized by the upregulation of *Col10a1* and *Mmp13*. Simultaneously, this inflammatory milieu sensitizes mesenchymal progenitors to BMP-2-mediated osteogenesis **(B)**, potentially via crosstalk between inflammatory and osteogenic pathways, resulting in the upregulation of osteogenic markers (*Runx2*, *OPN*, *OCN*, *ALP*) and the acceleration of pathological osteophyte formation.

In conclusion, our study identifies the inflammatory microenvironment as the critical determinant of BMP-2 function in OA (**Figure 6**). By converting a repair signal into a driver of osteophyte formation, inflammation fundamentally alters the trajectory of the disease. These insights argue for a paradigm shift in OA treatment: moving away from simple growth factor supplementation toward context-specific modulation of joint signaling networks. Future therapies must simultaneously dampen the inflammatory “noise” to allow the regenerative “signal” of BMP-2 to function correctly.

## Data Availability

The raw data supporting the conclusions of this article will be made available by the authors, without undue reservation.
