# Supplementary material for: The inflammatory microenvironment repurposes BMP-2 signaling to drive pathological osteophyte formation in osteoarthritis
Source: Front Immunol. 2026 Mar 25;17:1776833. doi: 10.3389/fimmu.2026.1776833 (PMC13105028; doi:10.3389/fimmu.2026.1776833)
Supplement: Supplementary file 1 [file Table1.docx]

**Supplementary Table 1. Baseline characteristics of study participants**

| **Variable** | **Control (n=10)** | **K-L 1–2 (n=10)** | **K-L 3–4 (n=10)** | **P value** |
| --- | --- | --- | --- | --- |
| Age (years) | 55.2 ± 6.3 | 56.8 ± 7.1 | 57.5 ± 6.8 | 0.78 |
| Female, n (%) | 6 (60%) | 7 (70%) | 6 (60%) | 0.89 |
| BMI (kg/m²) | 22.3 ± 1.8 | 23.1 ± 2.0 | 23.8 ± 2.2 | 0.32 |
| CRP (mg/L) | 2.4 ± 0.9 | 2.7 ± 1.0 | 2.9 ± 1.1 | 0.61 |
| ESR (mm/h) | 9.8 ± 3.0 | 10.5 ± 3.4 | 11.2 ± 3.2 | 0.56 |
| Diabetes, n (%) | 1 (10%) | 1 (10%) | 2 (20%) | 0.73 |
| Smoking history, n (%) | 2 (20%) | 2 (20%) | 3 (30%) | 0.85 |
